# Supplementary material for: Endometrial Cancer with and without Endometriosis: Clinicopathological Differences
Source: Cancers (Basel). 2023 Nov 29;15(23):5635. doi: 10.3390/cancers15235635 (PMC10705732; doi:10.3390/cancers15235635)
Supplement: Supplementary file 1 [file cancers-15-05635-s001.zip › cancers-2731586-supplementary.pdf]

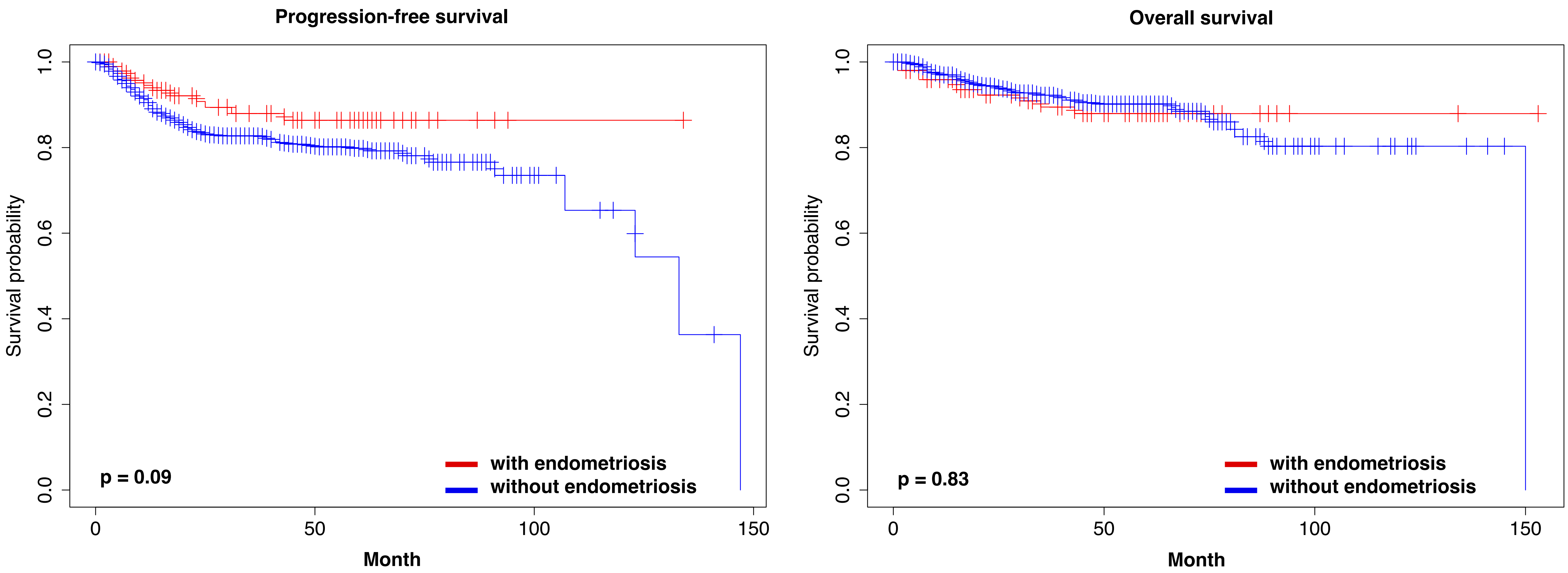

Supplementary Figure 1.
Kaplan Meier curves representing the survival in relation of all stages with and without endometriosis

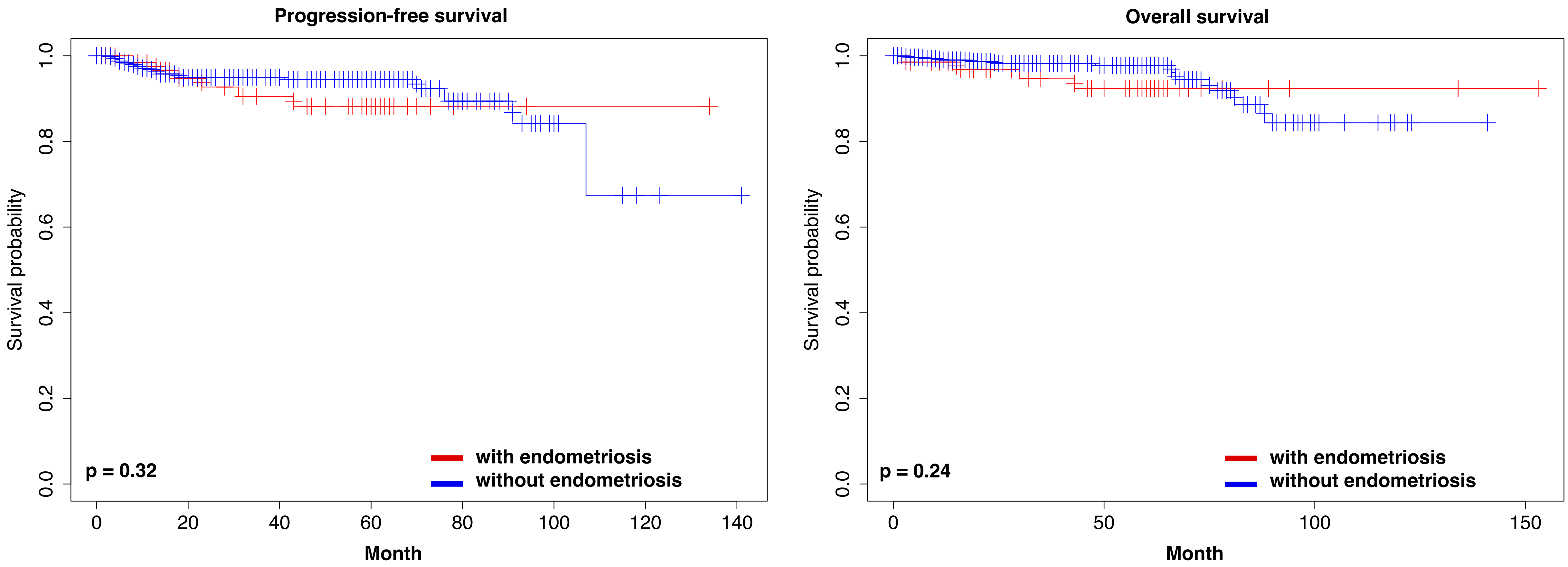

Supplementary Figure 2.
Kaplan Meier curves representing the survival in relation of stage IA with and without endometriosis
